# Dietary Interventions in Irritable Bowel Syndrome: A Systematic Review of Clinical Outcomes, Microbiota Changes, and Inflammatory Markers

**DOI:** 10.7759/cureus.70568

**Published:** 2024-09-30

**Authors:** Muhammad Shams, Junaid Ahmed, Aqsa Umar, Abdur Rehman, Komal Sohail, Bushra Javed, Raza Mustafa, FNU Payal, Abdullah Shehryar, Mustafa Khan

**Affiliations:** 1 Urology, Royal Bournemouth Hospital, Bournemouth, GBR; 2 Internal Medicine, Chandka Medical College, Larkana, PAK; 3 Internal Medicine, Mayo Hospital, Lahore, PAK; 4 Surgery, Mayo Hospital, Lahore, PAK; 5 Internal Medicine, Sahiwal Medical College, Sahiwal, PAK; 6 Internal Medicine, CMH Multan Institute of Medical Sciences, Multan, PAK; 7 Internal Medicine, Sligo University Hospital, Sligo, IRL; 8 Internal Medicine, Shaheed Mohtarma Benazir Bhutto Medical University, Larkana, PAK; 9 Internal Medicine, Allama Iqbal Medical College, Lahore, PAK; 10 General Surgery, Nishtar Medical University, Multan, PAK

**Keywords:** dietary interventions, gastrointestinal disorders, gut health, irritable bowel syndrome, nutrition therapy

## Abstract

This systematic review synthesizes findings from 12 studies to evaluate the effectiveness of dietary interventions in managing irritable bowel syndrome (IBS), with a focus on low-FODMAP (fermentable oligosaccharides, disaccharides, monosaccharides, and polyols) diets, probiotics, and prebiotics. The review rigorously follows the PRISMA (Preferred Reporting Items for Systematic Reviews and Meta-Analyses) guidelines and includes studies selected through comprehensive database searches. In adults diagnosed with IBS, this review assesses how effective dietary interventions, specifically low-FODMAP diets, probiotics, and prebiotics, are compared to standard management or placebo in improving clinical outcomes, modifying gut microbiota composition, and reducing inflammatory markers. Our analysis reveals that low-FODMAP diets consistently alleviate IBS symptoms and improve quality of life. However, the effectiveness of probiotics and prebiotics varies, with outcomes dependent on specific strains and individual patient microbiota profiles. The studies demonstrate significant improvements in gastrointestinal symptoms and microbiota composition, highlighting the potential of dietary strategies to beneficially modify gut health. However, the research points to the necessity of personalizing dietary approaches based on individual responses and microbiota profiles to optimize treatment efficacy. The risk of bias was assessed using the Cochrane risk-of-bias 2 tool for randomized controlled trials (RCTs) and the AMSTAR 2 tool for systematic reviews, with varying degrees of bias across the studies. This review identifies gaps in the long-term efficacy of these interventions and calls for more extensive trials to explore their sustained impacts. Our findings suggest that dietary management should be integrated into routine IBS treatment protocols and emphasize the need for further research to establish standardized dietary recommendations tailored to patient-specific characteristics.

## Introduction and background

Irritable bowel syndrome (IBS) remains one of the most common functional gastrointestinal disorders, affecting approximately 10-15% of the global population, with a significant burden on both individual quality of life and healthcare systems [1]. IBS is characterized by a complex constellation of symptoms, including abdominal pain, bloating, and altered bowel habits. Its pathophysiology is multifactorial and not completely understood, although it is thought to involve disruptions in the gut-brain axis, genetic predispositions, abnormalities in gut motility, visceral hypersensitivity, immune dysregulation, and psychosocial factors [2-4]. These diverse contributing factors make IBS a challenging condition to manage, with treatments often targeting symptom relief rather than addressing the underlying causes.

Recent advances have highlighted the role of diet in managing IBS symptoms, particularly through the modulation of gut microbiota, which has emerged as a significant factor in disease pathology and treatment [5]. The gut microbiota, comprising trillions of microorganisms, plays a critical role in digestion, immune function, and gut health. Alterations in the composition of the gut microbiota, or dysbiosis, have been linked to IBS, sparking interest in therapeutic approaches that focus on restoring a healthy microbiota balance.

Among the dietary interventions, the low-FODMAP (fermentable oligosaccharides, disaccharides, monosaccharides, and polyols) diet has gained attention for its ability to reduce symptoms by limiting the intake of fermentable substrates that cause osmotic fermentation in the colon, thereby reducing gas production and gastrointestinal distress [6]. Furthermore, probiotics and prebiotics, which aim to manipulate the gut microbiota by introducing beneficial bacterial strains or promoting their growth, have also been explored as potential treatments, with some studies showing improvements in symptoms and gut health [7]. However, despite promising results, there is significant heterogeneity in the findings of these studies, particularly regarding the efficacy of these interventions across different IBS subtypes (e.g., IBS with diarrhea, IBS with constipation), and their long-term effects remain unclear [8].

The existing literature on IBS management highlights certain gaps in the evidence base. For example, while several systematic reviews and meta-analyses have evaluated the effectiveness of the low-FODMAP diet, probiotics, and prebiotics individually, there is still inconsistency in the results, largely due to differences in study design, patient populations, and outcome measures. Moreover, these reviews often focus on short-term outcomes, leaving uncertainties about the long-term impact of dietary interventions on gut microbiota composition and inflammatory markers. There is also limited information on how individual microbiota profiles may influence response to treatment, pointing to the need for more personalized approaches in managing IBS. These gaps have motivated the need for a more comprehensive and detailed systematic review that synthesizes the current evidence while addressing these inconsistencies.

The primary objective of this systematic review is to rigorously evaluate and synthesize current research concerning the efficacy of dietary interventions, specifically low-FODMAP diets, probiotics, and prebiotics, in the management of IBS. By examining changes in clinical outcomes, alterations in gut microbiota, and modifications in inflammatory markers, this review aims to clarify the therapeutic potential of these dietary approaches. Through a detailed analysis of randomized controlled trials (RCTs) and meta-analyses, this review seeks to provide a critical assessment of the available evidence, identify gaps in the current knowledge, and offer recommendations for future research directions. This will aid clinicians in tailoring more effective, personalized dietary strategies for IBS patients, ultimately enhancing patient care and management strategies in clinical settings.

## Review

Materials and methods

Search Strategy

Our search strategy, developed in accordance with the Preferred Reporting Items for Systematic Reviews and Meta-Analyses (PRISMA) guidelines [9], focused on identifying relevant studies that examine the efficacy of dietary interventions for IBS. We conducted detailed searches across multiple databases, including PubMed, MEDLINE, Embase, the Cochrane Library, and Scopus, covering literature published from the inception of each database up to July 2024. The search strategy for each database was specifically tailored to its search capabilities. Keywords and Medical Subject Headings (MeSH) terms used in our search included "irritable bowel syndrome," "IBS," "low-FODMAP diet," "probiotics," "prebiotics," and "dietary management of IBS." Boolean operators ('AND', 'OR') were employed to combine these terms effectively. Filters were applied to limit results to studies published in English and focused on adult populations. The search strategy for PubMed, for instance, was as follows: ("irritable bowel syndrome"[MeSH Terms] OR "IBS") AND ("low FODMAP" OR "probiotics" OR "prebiotics") AND ("clinical trial" OR "randomized controlled trial"). Similar strategies were adapted for MEDLINE, Embase, Cochrane Library, and Scopus to ensure a comprehensive retrieval of relevant data across the databases.

To ensure the inclusion of the most relevant and recent data, we also reviewed the reference lists of all retrieved articles, checked clinical trial registries, and explored relevant conference proceedings for unpublished studies. This approach aimed to capture both published and grey literature, broadening the scope of our review and enhancing the robustness of our findings. Our search was limited to studies published in English and included randomized controlled trials, cohort studies, and meta-analyses to ensure a high standard of evidence in our systematic review.

Eligibility Criteria

To ensure the rigor and specificity of our systematic review, we established clear eligibility criteria based on the PICO framework (Population, Intervention, Comparison, Outcome). Our review focuses on peer-reviewed research articles that investigate dietary interventions for managing IBS. Specifically, we include clinical trials, RCTs, cohort studies, and meta-analyses that evaluate the effectiveness of interventions such as the low-FODMAP (fermentable oligosaccharides, disaccharides, monosaccharides, and polyols) diet, probiotics, and prebiotics in adults diagnosed with IBS (Population). These studies must provide detailed data on clinical outcomes (Outcome), including symptom management, changes in gut microbiota composition, and inflammatory markers, with comparisons to standard management or placebo where applicable (Comparison). We target studies conducted on adult populations diagnosed according to recognized criteria, such as the Rome III or IV criteria, which are standardized guidelines used to diagnose IBS based on specific symptom patterns. Articles published in English from the inception of the databases up to July 2024 are included to capture the most recent and comprehensive findings.

The exclusion criteria were carefully defined to refine the scope of our review. We excluded studies that focus on pediatric populations, as the response to dietary interventions can differ significantly between children and adults. Studies that do not explicitly assess the outcomes relevant to our review's objectives, such as IBS symptom relief, microbiota composition changes, and inflammatory marker alterations, are omitted. In addition, non-peer-reviewed articles, letters, editorials, and conference abstracts are excluded to maintain a high standard of evidence. Lastly, studies published in languages other than English are not considered to avoid potential inconsistencies from translation errors and ensure the precision and reliability of the data synthesized in our systematic review.

Data Extraction

For our systematic review of dietary interventions in managing IBS, we followed a structured data extraction process. Initially, articles were screened by two independent reviewers based on titles and abstracts to assess relevance. Articles were categorized as "relevant," "not relevant," or "potentially relevant." Full-text reviews were then conducted for "potentially relevant" articles to confirm eligibility according to our inclusion and exclusion criteria.

The data extraction itself was performed using a standardized form developed in Microsoft Excel to ensure uniformity and accuracy across the review process. Each selected article was meticulously analyzed by the reviewers, who independently extracted data on key variables such as study author(s), publication year, study design, sample size, intervention details, outcome measures, main results, and limitations. Discrepancies between reviewers were resolved through discussion or, if necessary, arbitration by a third reviewer. This methodical approach ensured that all relevant data were accurately captured, allowing for a robust synthesis and analysis aligned with the review’s objectives.

Data Analysis and Synthesis

Due to the heterogeneous nature of the studies examined regarding dietary interventions for IBS, a meta-analysis was deemed inappropriate. Instead, we adopted a qualitative synthesis approach to analyze the data, focusing on integrating and interpreting findings across different studies. This narrative synthesis allowed us to explore the effects of various dietary strategies on IBS symptoms, gut microbiota, and inflammatory markers comprehensively. By categorizing outcomes and identifying recurring themes and discrepancies among the studies, we provided a nuanced understanding of the efficacy of interventions like low-FODMAP diets, probiotics, and prebiotics. This method not only highlighted the complexities of dietary effects on IBS but also pinpointed research gaps and potential areas for future investigation.

Results

Study Selection Process

**Figure 1:**
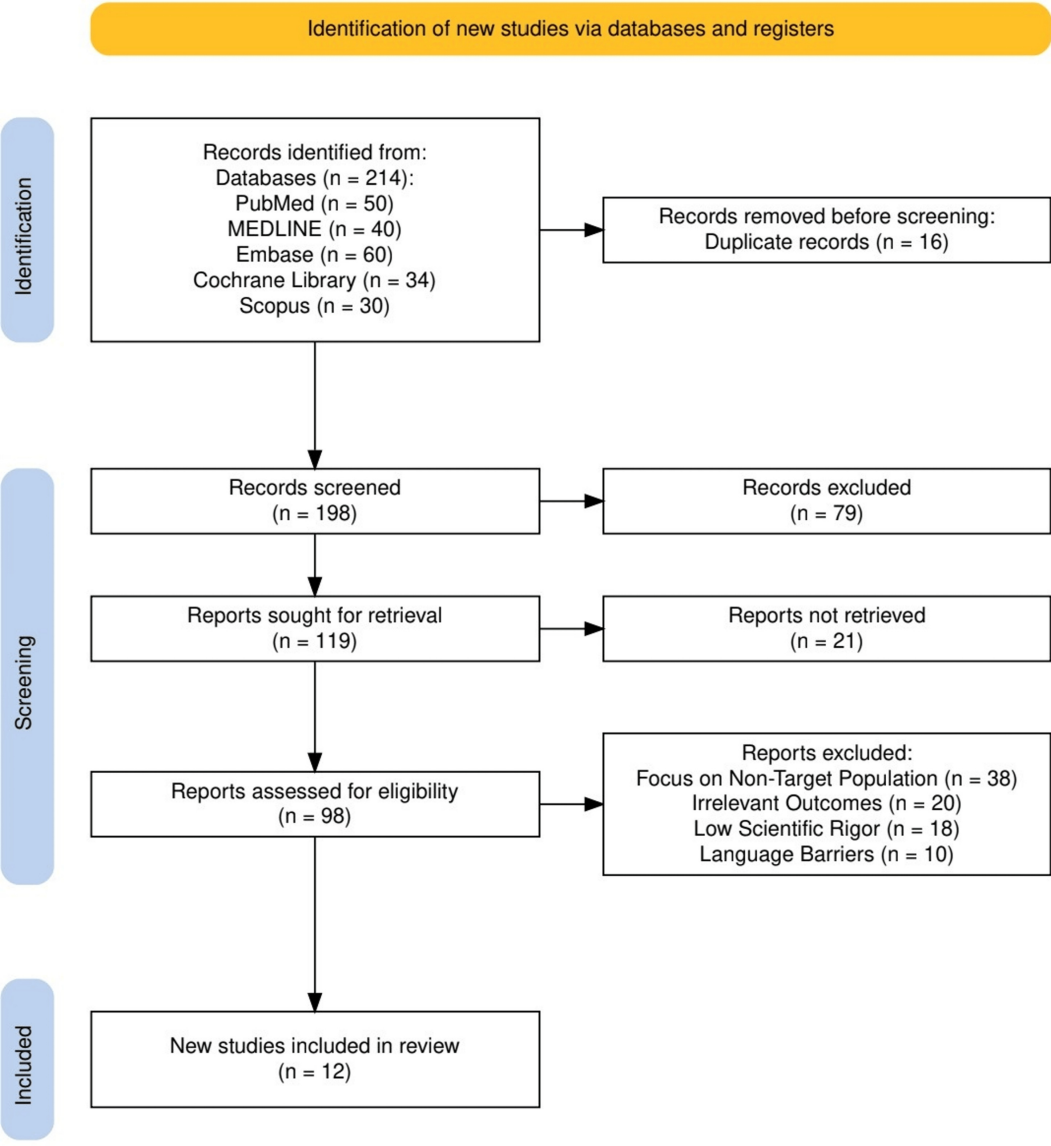
The PRISMA flowchart representing the study selection process. PRISMA: Preferred Reporting Items for Systematic Reviews and Meta-Analyses

The study selection process for our systematic review was meticulously organized to ensure the inclusion of comprehensive and relevant studies. Initially, we identified a total of 214 records from a variety of databases, including PubMed (50 records), MEDLINE (40 records), Embase (60 records), the Cochrane Library (34 records), and Scopus (30 records). After removing 16 duplicates, we screened 198 articles based on their titles and abstracts. This screening led to the exclusion of 79 records that did not meet preliminary inclusion criteria, such as relevance to dietary interventions in IBS or providing sufficient detail in the abstracts. We then sought to retrieve 119 full-text articles for a more detailed evaluation, of which 21 were not available. The remaining 98 reports underwent a thorough review against our specified inclusion and exclusion criteria. This phase resulted in the exclusion of an additional 86 reports, primarily due to their failure to directly address the research questions or the lack of necessary data. Ultimately, 12 studies were deemed eligible and included in the review, forming the basis for our comprehensive analysis of dietary interventions in the management of IBS. A summary of the study selection process is given in Figure 1.

Characteristics of the Selected Studies

The studies selected for our systematic review span a range of methodologies and focus areas within the scope of dietary interventions for IBS. These investigations encompass RCTs, double-blind crossover trials, and systematic reviews with meta-analysis, highlighting a robust approach to exploring the efficacy of dietary strategies in IBS management. The interventions examined include low-FODMAP (fermentable oligosaccharides, disaccharides, monosaccharides, and polyols) diets, probiotics, prebiotics, and their impact on different populations, including both adult and pediatric patients, across various IBS subtypes. Key outcomes from these studies consistently indicate improvements in IBS symptoms such as reduced severity and enhanced quality of life, alongside beneficial changes in gut microbiota. The evidence supports the short-term efficacy of low-FODMAP diets in reducing gastrointestinal discomfort and suggests variable effectiveness of probiotics dependent on strain specificity. The findings collectively underscore the potential of personalized dietary management in IBS treatment, emphasizing the importance of tailoring interventions to individual dietary responses and microbiota profiles to optimize therapeutic outcomes. A summary of the selected studies is given in Table 1.

**Table 1:** A summary of the included studies in this systematic review. IBS: irritable bowel syndrome; RCT: randomized controlled trial; FMT: fecal microbiota transplantation; IBS-D: irritable bowel syndrome with diarrhea; FODMAP: fermentable oligosaccharides, disaccharides, monosaccharides, and polyols; LFD: low-FODMAP diet; TDA: traditional dietary advice; LF-GFD: low-FODMAP-gluten-free diet; FOS: fructooligosaccharide

Authors	Study year	Purpose	Methods	Results	Conclusions	Risk of bias (method employed)
Anne-Sophie van Lanen et al. [10]	2021	Investigate low-FODMAP diet effects on IBS symptoms, life quality, nutrition, and gut microbiome	Systematic search and meta-analysis with random effects models	Reduced IBS severity and improved quality of life	Low-FODMAP diet is effective but requires more research on long-term effects.	Low risk of bias (Cochrane Risk of Bias Tool for Systematic Reviews)
Ruggiero Francavilla et al. [11]	2019	Assess the efficacy and safety of a probiotic mixture in celiac patients with IBS symptoms on a gluten-free diet	Double-blind, randomized, placebo-controlled multicenter study over six weeks	Significant improvement in IBS symptoms and increased beneficial gut bacteria	Probiotics are effective in improving IBS-type symptoms in celiac disease patients.	Low risk of bias (Cochrane Risk of Bias 2 (RoB 2) Tool for Randomized Controlled Trials)
Muhammad Ali Khan et al. [12]	2015	Evaluate low-FODMAP diet as a treatment strategy for IBS	Review of trials and studies on low-FODMAP diet's mechanism and efficacy	Promising results in symptom reduction but significant changes in gut microbiota	Effective in reducing symptoms; impacts on gut health need further study.	Moderate risk of bias (AMSTAR 2 Tool for Systematic Reviews)
Soo Liang Ooi et al. [13]	2019	Assess current evidence on probiotics, prebiotics, and low-FODMAP diet efficacy in IBS treatment	Systematic review of reviews and meta-analyses	Mixed results on probiotics and prebiotics; low-FODMAP diet improves symptoms	Probiotics and low-FODMAP diet are effective under supervision; prebiotics lack clinical evidence.	Low risk of bias (Cochrane Risk of Bias Tool for Systematic Reviews)
Dania Schumann et al. [14]	2018	Meta-analyze the low-FODMAP diet's effects on IBS symptom severity, quality of life, and safety	Screening of databases and inclusion of RCTs comparing LFD to other diets	Improvements in symptoms and quality of life; reduction in certain gut bacteria	LFD shows short-term efficacy and safety; long-term effects require further investigation.	Low risk of bias (Cochrane Risk of Bias Tool for Systematic Reviews)
Magdy El-Salhy et al. [15]	2021	Investigate sex differences in response to FMT in IBS patients	Study of 164 IBS patients from a previous RCT, analyzing severity, quality of life, and microbial changes	No sex differences in overall response; females with IBS-D had better outcomes.	No general sex differences, but notable female responses in IBS-D.	Moderate risk of bias (Cochrane Risk of Bias 2 (RoB 2) Tool for Randomized Controlled Trials)
Yawen Zhang et al. [16]	2021	Compare low-FODMAP diet and traditional dietary advice in Chinese IBS-D patients	Randomization of 108 patients to diets, assessment of symptoms, and microbiota changes	Similar improvement rates; earlier symptom relief with low-FODMAP	LFD and TDA are both effective; LFD offers quicker symptomatic relief.	Low risk of bias (Cochrane Risk of Bias 2 (RoB 2) Tool for Randomized Controlled Trials)
Kaveh Naseri et al. [17]	2021	Study low FODMAP-gluten-free diet's impact on IBS symptoms, microbiota diversity, and fecal calprotectin	A six-week clinical trial with 42 IBS patients, using symptom scoring and microbiota analysis	Significant symptom improvement and microbiota normalization	LF-GFD improves symptoms and gut health; further studies are needed for long-term efficacy.	Moderate risk of bias (Cochrane Risk of Bias 2 (RoB 2) Tool for Randomized Controlled Trials)
B P Chumpitazi et al. [18]	2015	Evaluate low-FODMAP diet efficacy in childhood IBS and its association with gut microbiome	Double-blind, crossover trial with dietary intervention, microbiome, and symptom analysis	Less abdominal pain with low-FODMAP; specific microbiome changes linked to diet response	Effective in reducing childhood IBS symptoms; microbiome biomarkers predict response.	Moderate risk of bias (Cochrane Risk of Bias 2 (RoB 2) Tool for Randomized Controlled Trials)
T N Hustoft et al. [19]	2017	Compare the effects of low-FODMAP and high-FOS diets on IBS symptoms and microbiota	A nine-week study with supplement intervention and comprehensive symptom and microbiota profiling	Improvement with low-FODMAP; specific bacterial and cytokine changes noted	Low-FODMAP alleviates symptoms and impacts gut microbiota; FOS has limited effect on cytokines or microbiota.	Moderate risk of bias (Cochrane Risk of Bias 2 (RoB 2) Tool for Randomized Controlled Trials)
María Ortiz-Lucas et al. [20]	2013	Assess the efficacy of various probiotic species on IBS symptoms	Meta-analysis of trials comparing probiotics to placebo	Specific probiotics improve pain, distension, and flatulence scores	Probiotics provide symptom relief; effects vary by species and need further investigation.	Moderate risk of bias (AMSTAR 2 Tool for Systematic Reviews)
D B A Silk et al. [21]	2009	Examine the efficacy of a trans-galactooligosaccharide prebiotic on IBS symptoms and gut microflora	A 12-week controlled clinical trial with dosage variation, monitoring symptoms, and microbiota changes.	Increased bifidobacteria and improvements in stool consistency, flatulence, bloating, and psychological symptoms	Prebiotic stimulates beneficial bacteria and effectively alleviates IBS symptoms.	Moderate risk of bias (Cochrane Risk of Bias 2 (RoB 2) Tool for Randomized Controlled Trials)

Discussion

Our systematic review rigorously analyzed the efficacy of various dietary interventions in the management of IBS, encompassing a total of 12 foundational studies. Key findings consistently highlighted the significant impact of low-FODMAP diets, probiotics, and prebiotics on alleviating IBS symptoms, improving the quality of life, and modifying gut microbiota composition. Specifically, low-FODMAP diets were frequently associated with reductions in gastrointestinal discomfort and improvements in overall well-being [22]. Probiotics and prebiotics, meanwhile, demonstrated positive effects on gut health, contributing to increased levels of beneficial bacteria and decreased inflammation, although the results varied based on specific strains and formulations [23]. These findings address our initial research hypotheses positing that tailored dietary strategies can significantly influence clinical outcomes in IBS patients, providing a clear link between diet modification and symptomatic relief.

This comprehensive evaluation highlights the complexity and heterogeneity of dietary responses in IBS, suggesting that while dietary interventions hold promise, their effectiveness is influenced by individual patient characteristics including microbiota composition and dietary adherence [24]. The analysis not only confirms the potential of these dietary approaches as part of a broader IBS management strategy but also highlights the necessity for personalized dietary recommendations to maximize therapeutic outcomes. This synthesis of data provides a pivotal foundation for further discussion on the integration of these findings into clinical practice and ongoing research [25].

The findings of our systematic review are largely consistent with existing literature that underscores the benefits of dietary interventions in managing IBS. For instance, several studies within our review reported significant improvements in IBS symptoms with low-FODMAP diets, aligning with findings from prior meta-analyses such as Marsh et al. (2016) [26], which also documented a reduction in symptom severity among IBS patients adhering to low-FODMAP diets. However, while our review highlighted some variability in the effectiveness of probiotics, this contrasts with more definitive positive outcomes noted in studies [27], which found consistent benefits of specific probiotics strains for overall IBS symptom management. These discrepancies may be attributed to differences in study designs, populations, probiotic strains used, and duration of treatment across studies.

Furthermore, our review extends the existing knowledge base by examining the interplay between diet, gut microbiota, and inflammatory markers in IBS, an area that has received less attention in earlier reviews. While we noted improvements in gut microbiota composition and inflammatory markers in response to dietary interventions, these results were not uniformly observed across all included studies, suggesting a complex interaction influenced by individual patient microbiomes and dietary compliance. This complexity is echoed in the work of Chumpitazi et al. [18], which found that responses to dietary interventions could vary significantly based on baseline microbiota profiles, pointing to the need for personalized dietary management strategies in IBS treatment. Such findings indicate a move toward more individualized therapeutic approaches in the gastroenterological field, highlighting the importance of tailoring treatments to the specific biological and clinical profiles of IBS patients [28].

The outcomes of our systematic review have significant theoretical implications for the understanding and management of IBS. By demonstrating the efficacy of dietary interventions such as low-FODMAP diets, probiotics, and prebiotics in modulating symptoms and altering gut microbiota, our findings reinforce and expand the gut-brain axis theory in IBS. This theory posits that a bidirectional communication exists between the gastrointestinal tract and the brain, influencing both physical and psychological aspects of the disease [29]. Our analysis supports this model by showing how dietary modifications can lead to changes in gut microbiota composition and subsequent symptom alleviation, possibly through mechanisms involving short-chain fatty acids and inflammatory cytokines. These insights contribute to a deeper understanding of the pathophysiology of IBS and suggest that dietary management could play a crucial role in breaking the cycle of dysbiosis and inflammation thought to exacerbate the condition. Consequently, our review not only aligns with existing gastrointestinal models but also enhances them by highlighting the integral role of diet and microbial health in managing chronic gastrointestinal disorders [30].

The practical implications of our systematic review are profound, offering clear guidance for clinicians and dietitians managing IBS. The demonstrated efficacy of low-FODMAP diets, probiotics, and prebiotics in alleviating IBS symptoms provides a strong basis for these dietary strategies to be integrated into standard IBS management protocols. Clinicians are encouraged to consider individual dietary counseling as part of the treatment plan, which includes assessing patients' dietary habits, symptom triggers, and tolerance to specific foods. Furthermore, given the variability in response to probiotics, healthcare providers should consider personalized approaches, possibly guided by gut microbiota profiling, to identify the most beneficial probiotic strains for individual patients. For policymakers, these findings highlight the importance of supporting and funding dietary management programs as a cost-effective approach to reduce the burden of IBS on healthcare systems. In addition, the connection between diet, gut microbiota, and IBS symptoms emphasized in our review suggests that further research should focus on longitudinal studies to explore the long-term effects of dietary interventions and their impact on life quality among IBS patients. This would aid in refining dietary recommendations and tailoring more effective, patient-specific therapeutic options [31,32].

Our systematic review is underpinned by several methodological strengths that enhance the credibility and reliability of our findings. Foremost, the adherence to the PRISMA guidelines ensured a structured and transparent approach, facilitating reproducibility and rigor in our review process. The comprehensive search strategy across multiple databases, including PubMed, MEDLINE, and Embase, allowed for an extensive collection of relevant studies, minimizing the risk of publication bias. In addition, the inclusion of diverse dietary interventions such as low-FODMAP diets, probiotics, and prebiotics provided a holistic view of the available treatments for IBS, enhancing the applicability of our conclusions to various patient populations.

However, our review is not without limitations. The heterogeneity in study designs, populations, and outcome measures among the included studies posed challenges in drawing uniform conclusions, which may limit the generalizability of our findings. Furthermore, the restriction to articles published in English could have omitted relevant studies conducted in other languages, potentially introducing language bias. The exclusion of gray literature and unpublished studies might also limit the comprehensiveness of our analysis. Future research should aim to include a broader range of studies, possibly through meta-analyses that can quantify the effects more precisely and explore the relationships between diet, microbiota changes, and IBS symptoms in different subpopulations and settings.

Building upon the insights gained from our systematic review, several areas warrant further investigation to deepen the understanding of dietary interventions in IBS. Future research should focus on longitudinal studies to explore the long-term efficacy and safety of low-FODMAP diets, probiotics, and prebiotics. There is also a critical need for larger, multicentric randomized controlled trials to assess the variability in individual responses to these dietary interventions, potentially exploring genetic, environmental, or microbiome-related factors that influence treatment outcomes. In addition, studies should aim to integrate advanced technologies such as metagenomic sequencing to provide more detailed insights into how these dietary strategies alter gut microbiota composition and function. Addressing these gaps will not only enhance our understanding of the mechanisms underlying the dietary management of IBS but also improve the personalization of treatment strategies, ultimately leading to better patient outcomes [33].

## Conclusions

Our systematic review underscores the significant potential of dietary interventions, namely, low-FODMAP diets, probiotics, and prebiotics, as effective strategies for managing IBS. These approaches not only alleviate symptoms but also contribute to understanding the complex interactions within the gut microbiota and its influence on the condition. By integrating these dietary strategies into routine clinical practice, healthcare providers can offer more personalized and effective treatment plans. Moreover, our findings highlight the importance of dietary management as a cornerstone of IBS treatment, which, when appropriately tailored, can significantly enhance patient quality of life. We encourage the scientific and medical communities to continue exploring the intricate dynamics between diet and gut health, pushing the boundaries of what is currently known and expanding the therapeutic possibilities for IBS patients worldwide.

## References

[1] Weaver KR, Melkus GD, Henderson WA (2017). Irritable bowel syndrome. Am J Nurs.

[2] Saha L (2014). Irritable bowel syndrome: pathogenesis, diagnosis, treatment, and evidence-based medicine. World J Gastroenterol.

[3] Mayer EA, Ryu HJ, Bhatt RR (2023). The neurobiology of irritable bowel syndrome. Mol Psychiatry.

[4] Chong PP, Chin VK, Looi CY, Wong WF, Madhavan P, Yong VC (2019). The microbiome and irritable bowel syndrome - a review on the pathophysiology, current research and future therapy. Front Microbiol.

[5] Kim MY, Choi SW (2021). Dietary modulation of gut microbiota for the relief of irritable bowel syndrome. Nutr Res Pract.

[6] Morariu ID, Avasilcai L, Vieriu M (2023). Effects of a low-FODMAP diet on irritable bowel syndrome in both children and adults—a narrative review. Nutrients.

[7] Roy S, Dhaneshwar S (2023). Role of prebiotics, probiotics, and synbiotics in management of inflammatory bowel disease: current perspectives. World J Gastroenterol.

[8] Wang Y, Ma W, Mehta R (2023). Diet and gut microbial associations in irritable bowel syndrome according to disease subtype. Gut Microbes.

[9] Page MJ, McKenzie JE, Bossuyt PM (2021). The PRISMA 2020 statement: an updated guideline for reporting systematic reviews. BMJ.

[10] van Lanen AS, de Bree A, Greyling A (2021). Efficacy of a low-FODMAP diet in adult irritable bowel syndrome: a systematic review and meta-analysis. Eur J Nutr.

[11] Francavilla R, Piccolo M, Francavilla A (2019). Clinical and microbiological effect of a multispecies probiotic supplementation in celiac patients with persistent ibs-type symptoms: a randomized, double-blind, placebo-controlled, multicenter trial. J Clin Gastroenterol.

[12] Khan MA, Nusrat S, Khan MI, Nawras A, Bielefeldt K (2015). Low-FODMAP diet for irritable bowel syndrome: is it ready for prime time?. Dig Dis Sci.

[13] Ooi SL, Correa D, Pak SC (2019). Probiotics, prebiotics, and low FODMAP diet for irritable bowel syndrome - what is the current evidence?. Complement Ther Med.

[14] Schumann D, Klose P, Lauche R, Dobos G, Langhorst J, Cramer H (2018). Low fermentable, oligo-, di-, mono-saccharides and polyol diet in the treatment of irritable bowel syndrome: a systematic review and meta-analysis. Nutrition.

[15] El-Salhy M, Casen C, Valeur J, Hausken T, Hatlebakk JG (2021). Responses to faecal microbiota transplantation in female and male patients with irritable bowel syndrome. World J Gastroenterol.

[16] Zhang Y, Feng L, Wang X (2021). Low fermentable oligosaccharides, disaccharides, monosaccharides, and polyols diet compared with traditional dietary advice for diarrhea-predominant irritable bowel syndrome: a parallel-group, randomized controlled trial with analysis of clinical and microbiological factors associated with patient outcomes. Am J Clin Nutr.

[17] Naseri K, Dabiri H, Rostami-Nejad M (2021). Influence of low FODMAP-gluten free diet on gut microbiota alterations and symptom severity in Iranian patients with irritable bowel syndrome. BMC Gastroenterol.

[18] Chumpitazi BP, Cope JL, Hollister EB (2015). Randomised clinical trial: gut microbiome biomarkers are associated with clinical response to a low FODMAP diet in children with the irritable bowel syndrome. Aliment Pharmacol Ther.

[19] Hustoft TN, Hausken T, Ystad SO, Valeur J, Brokstad K, Hatlebakk JG, Lied GA (2017). Effects of varying dietary content of fermentable short-chain carbohydrates on symptoms, fecal microenvironment, and cytokine profiles in patients with irritable bowel syndrome. Neurogastroenterol Motil.

[20] Ortiz-Lucas M, Tobías A, Saz P, Sebastián JJ (2013). Effect of probiotic species on irritable bowel syndrome symptoms: a bring up to date meta-analysis. Rev Esp Enferm Dig.

[21] Silk DB, Davis A, Vulevic J, Tzortzis G, Gibson GR (2009). Clinical trial: the effects of a trans-galactooligosaccharide prebiotic on faecal microbiota and symptoms in irritable bowel syndrome. Aliment Pharmacol Ther.

[22] Dionne J, Ford AC, Yuan Y (2018). A systematic review and meta-analysis evaluating the efficacy of a gluten-free diet and a low FODMAPs diet in treating symptoms of irritable bowel syndrome. Am J Gastroenterol.

[23] Ji J, Jin W, Liu SJ, Jiao Z, Li X (2023). Probiotics, prebiotics, and postbiotics in health and disease. MedComm (2020).

[24] Jayasinghe M, Karunanayake V, Mohtashim A (2024). The role of diet in the management of irritable bowel syndrome: a comprehensive review. Cureus.

[25] Algera J, Colomier E, Simrén M (2019). The dietary management of patients with irritable bowel syndrome: a narrative review of the existing and emerging evidence. Nutrients.

[26] Marsh A, Eslick EM, Eslick GD (2016). Does a diet low in FODMAPs reduce symptoms associated with functional gastrointestinal disorders? A comprehensive systematic review and meta-analysis. Eur J Nutr.

[27] Dale HF, Rasmussen SH, Asiller ÖÖ, Lied GA (2019). Probiotics in irritable bowel syndrome: an up-to-date systematic review. Nutrients.

[28] Anand K, Khatib MN (2024). Causative factors, clinical manifestations, and therapeutic strategies for irritable bowel syndrome. Cureus.

[29] Black CJ, Ford AC (2021). Best management of irritable bowel syndrome. Frontline Gastroenterol.

[30] Yan J, Wang L, Gu Y, Hou H, Liu T, Ding Y, Cao H (2022). Dietary patterns and gut microbiota changes in inflammatory bowel disease: current insights and future challenges. Nutrients.

[31] Bertin L, Zanconato M, Crepaldi M (2024). The role of the FODMAP diet in IBS. Nutrients.

[32] Nanayakkara WS, Skidmore PM, O'Brien L, Wilkinson TJ, Gearry RB (2016). Efficacy of the low FODMAP diet for treating irritable bowel syndrome: the evidence to date. Clin Exp Gastroenterol.

[33] Radziszewska M, Smarkusz-Zarzecka J, Ostrowska L (2023). Nutrition, physical activity and supplementation in irritable bowel syndrome. Nutrients.

